# A New Approach in Surface Modification and Surface Hardening of Aluminum Alloys Using Friction Stir Process: Cu-Reinforced AA5083

**DOI:** 10.3390/ma13061278

**Published:** 2020-03-12

**Authors:** Ioannis G. Papantoniou, Angelos P. Markopoulos, Dimitrios E. Manolakos

**Affiliations:** Laboratory of Manufacturing Technology, School of Mechanical Engineering, National Technical University of Athens, Heroon Polytechniou 9, 15780 Athens, Greece; ipapanto@central.ntua.gr (I.G.P.); manolako@central.ntua.gr (D.E.M.)

**Keywords:** surface modification, friction stir processing, in-situ composite, surface composite, AA5083, FSP

## Abstract

In the current study, a new approach for surface modification and surface hardening of aluminum alloys is developed. The method is based on the logic of in-situ reinforcing FSP strategies. The novelty of the proposed process is the application of a bulk reinforcing metallic material instead of metallic powders. The FSP was carried out on aluminum alloy AA5083—thick plates. A thin sheet of pure copper (cross-section 4 × 0.8 mm^2^) was placed in a machined groove on the upper surface of the aluminum plate, and both materials were FSPed together. Samples with one, two and three FSP passes were manufactured respectively. Results indicate that the copper thin sheet was successfully integrated in the AA5083 stir zone. By increasing the FSP passes, almost all copper was integrated in the stir zone, mainly in the form of coper-based micron-sized intermetallic particles, and secondly, by copper diffusion in the AA5083 matrix. Due to the presence of complex intermetallic compounds created by the high heat input and intense plastic deformation, the hardness inside the stir-zone was found highly increased from 77 to 138 HV.

## 1. Introduction

Today, aluminum and its alloys are used in different areas of manufacturing and technology (e.g., automotive and aerospace) [1]. Modern industries have been aggressively pushing the limits of aluminum alloys in strength, damage tolerance and corrosion resistance fronts to develop strong and tough aluminum alloys for various parts to increase the overall efficiency [2,3]. The aluminum alloy 5xxx series is widely utilized in marine applications, such as ship hulls. AA5083 is considered one of the well-known representatives of the 5××× series, with many applications in aircraft, marine structural parts and automobiles [4]. Welding of aluminum alloys is important for fabricating structural constructions and mechanical fabrications such as aircraft and marine vessels. However, welding exhibits problems and can be challenging in many cases. Indicative welding defects common to aluminum include incomplete fusion, hot cracking and porosity [5]. 

Friction stir welding (FSW) is a solid-phase welding process giving good quality butt and lap joints and is being widely considered by the modern aerospace and automotive industries for high-performance, structurally-demanding applications. The peak welding temperature can be limited to 80% of the melting temperature of the base metal [6]. Therefore, this process can be considered as a hot working process. Thus, the FSW process has proved to be ideal for creating high quality welds in several materials, including those which are extremely difficult to weld by conventional fusion processes [7,8]. Furthermore, FSW is proven to avoid severe distortions, and the generated residual stresses are found to be particularly low, compared to traditional welding processes [9].

Surface modifications of engineering components that comprise surface interactions are of major importance in the industries nowadays. The surfaces of engineering components can be traditionally protected by different methods (e.g., protective coatings and induction hardening). During the last decade, surface composites have been emerging as an effective way to improve the surface hardness and protect the components against wear and to reduce friction. One of the most important surface composite manufacturing processes is the friction stir process (FSP) [10]. FSP is a surface modifying technique which involves the generation of friction heat and intense plastic flow. FSP was developed from the basic principles of FSW. Owing to its high mixing ability, methods based on FSP have been developed for the incorporation of reinforcing particles into the modified surface (stir-zone) to further improve the hardness of the base materials [11]. The particles used as reinforcing fillers are mostly either ceramic (e.g., Al_2_O_3_) or carbide (e.g., TiC, SiC or WC) powders. Kurt et al. [12] incorporated SiC particles into cold-rolled plates of AA1050 using FSP; the hardness values were increased from 67 HV (plain specimen) to 80 HV, and the bending strength was increased from 60 MPa to 84 MPa. Navazani et al. [13] used FSP for the introduction and fine dispersion of nano-ZrO_2_ particles in AZ31 alloy; the obtained specimens introduced enhanced hardness values from 55 HV for the plain material to 87 HV for the two FSP pass reinforced one. The yield stress and the ultimate tensile strength were found increased in the FSPed specimens; on the other hand, the toughness was decreased. The change in the mechanical properties was found attributed mainly to the refinement of the microstructure. Mirjavadi et al. [14] manufactured AA5083/ZrO2 nanocomposites through FSP process. The hardness values were gradually increased by increasing the FSP passes due to constant refinement of the microstructure; 130 HV hardness were achieved for eight FSP passes. Another approach in surface reinforcing using FSP is in-situ reinforcing. In-situ approach of surface reinforcing involves synthesizing desirable reinforcements during processing itself. In-situ composites present many benefits, such as reinforcement-matrix interfaces free of defects, thermodynamically stable reinforcements, improved compatibility and higher bonding strength between the reinforcements and the matrix [15,16]. 

Based on the above, a key limitation is identified and concerns the fact that aluminum-based and aluminum-alloy-based metal matrix composites, formed during friction stir process by an in-situ reaction, are of high interest and have not been well researched. Thus, in the current study, a new approach in applying friction stir process for fabricating in-situ composites is presented. The method starts with the machining of a shallow groove in the surface of the base material. The groove is then filled with a thin sheet of the reinforcing metal, and then multiple FSP passes are carried out for the integration of the reinforcing metal into the FSPed stir-zone. The logic of the process is that: (a) the intense stirring action of the process will integrate the thin soft sheet of the reinforcing metal in the form of small particles inside the stir zone; (b) due to the high heat input of the FSP process, the integrated reinforcing particles are expected to form in situ intermetallic compounds, and these intermetallics will act as secondary phase reinforcements to the matrix; (c) at the same time, diffusion of the reinforcing metal in the matrix could lead to a solid solution matrix that is mechanically tougher. Thus, in the present study, plates of the non-heat treatable aluminum alloy AA5083-H111 were used as the base material. As reinforcing material, a thin sheet of pure copper was used; the reinforcements in the aluminum matrix were synthesized by in-situ chemical reactions between copper and AA5083 during the FSP process. Copper was used because it is one of the few elements that has relatively high solubility in aluminum. Furthermore, Al–Cu systems are being extensively studied due to their potential applications at ambient to elevated temperatures [17]. Specimens with different numbers of FSP passes were successfully created and analyzed by the means of optical microscopy (OM), scanning electron microscopy (SEM), atomic force microscopy (AFM) and X-ray diffraction (XRD) techniques. Finally, the macrohardness distribution was calculated, evaluated and correlated to the microstructural outcomes.

## 2. Materials and Methods

### 2.1. Materials

As the base material, 6 mm thick plates of aluminum alloy AA5083-H111 (in work hardened condition) were used. Aluminum alloy AA5083 is a non-heat treatable aluminum alloy widely used in the transportation, marine and chemical industries. It presents excellent weldability and formability, mild strength and mild corrosion resistance [18]. A spectrometer was used to verify the chemical constituents of the AA5083 plates. The analytical chemical composition is given in Table 1. The given values are the mean values of three independent measurements. A thin sheet of pure copper with cross-section 4 mm width and 0.8 mm thickness was used as the reinforcing material. Furthermore, a thin sheet of pure aluminum with cross-section 4 mm width and 0.6 mm thickness was used to cover the copper sheet (the usage of this layer is analytically explained in Section 2.2). 

The implementation of a copper strip instead of powder minimizes the volume of reinforcing material needed (and the groove’s dimensions), reduces the air inserted into the stir zone and introduces a more economical process of surface composite fabrication.

### 2.2. Friction Stir Process

**Figure 1:**
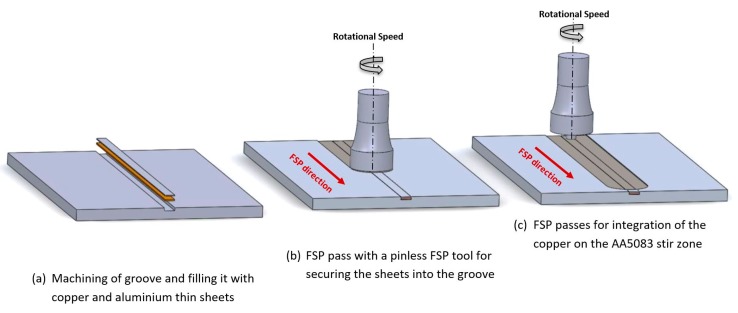
FSP-based manufacturing process of Cu-reinforced AA5083 specimens; (**a**) filling with thin sheets, (**b**) pass with pinless tool and (**c**) FSP passes with tool with pin.

Figure 1 illustrates the methodology followed in the manufacturing process. Initially, a groove with cross-section of 4 mm width and 1.4 mm deep was machined in the aluminum plate. The copper thin sheet was placed in the bottom of the groove and the pure aluminum sheet was placed above (Figure 1a). The groove was covered/closed, using a pinless tool, by a single FSP pass (Figure 1b). This stage was necessary in order to create a surface aluminum layer that keeps the thin copper sheet stable inside the groove during the next FSP stage. The parameters used for the pinless FSP pass were 1000 rpm rotational speed combined with 120 mm/min transverse speed. Preliminary experiments necessitated the use of the upper thin aluminum sheet because of the direct contact of the FSW tools with the copper led to aluminum/copper intermetallic material getting bonded on the steel FSW tool due to high heat input and intense plastic deformation. The adhesion of the intermetallic compounds to the FSP tool threated pin resulted in wormhole type defects and the adhesion in the shoulder resulted in surface morphology defects. The addition of a thin aluminum sheet that covered the copper eliminated the adhesion of compounds to the FSP tool.

Initially, experiments were conducted with the intention of identifying the optimum parameters that result in a FSPed stir zone consisting of good material mixing and no defects. The optimum operational parameters were obtained for 1000 rpm rotational speed (maximum of the FSW machine) combined with 13 mm/min transverse speed (lowest of the used FSW machine). Those parameters, due to the high heat input (high weld pitch) and the intense plastic deformation, were projected according to literature to present the optimum material mixing for AA5083 friction stir processing [19,20]. 

Thus, after the stage of closing the groove with the pinless tool (Figure 1b), FSP passes were carried out in the same direction, successively and without allowing the samples between the FSP passes to cool down to room temperature (Figure 1c). Furthermore, in order to study the influence of the number of FSP passes on the integration of copper in the stir-zone; one, two and three passes were performed respectively.

The FSP experiments were carried out by the use of a modified milling machine. The friction stir welding/processing tool was made from heat-treated tool steel. The shoulder of the tool was flat with a diameter of 22 mm. The pin of the tool was cylindrical with a right-handed thread, 4 mm diameter and 4 mm height. 

### 2.3. Microstructural Characterization

Initially, a metallographic specimen preparation was performed. The FSPed specimens were cut perpendicularly to the direction of the FSP. Then the specimens were mounted in epoxy resin, and their surfaces were grinded and polished, reaching a final polishing stage of 0.05 μm colloidal silica suspension. The samples were then treated for a short period of time in an ultrasonic bath in order to remove the remaining particles from the polishing stage. Finally, the samples were etched by a soft Keller's reagent for ten seconds.

After the metallographic specimen preparation, an analytical microstructural characterization was performed on the stir zone of the friction stir processed specimens. Firstly, the specimens were observed using optical microscopy (OM) methods. An optical stereoscope Leica MZ6 (Wetzlar, Hesse, Germany) was used for the macroscopic observation of the FSPed stir zones, and an optical microscope Leica DMILM was used for investigation of the material flow inside the stir zone. The embedded copper-based intermetallic particles were further characterized using a scanning electron microscope (SEM) equipped with energy-dispersive spectroscopy (EDS) detector at an accelerated voltage of 20 kV. Furthermore, in order to observe the surface topography characteristics, atomic force microscopy (AFM) experiments were carried out. Finally, an X-ray diffraction (XRD) study was performed in order to investigate the phases formed in the friction-stir-processed composite stir-zone.

### 2.4. Macrohardness Distribution Evaluation

The microstructural observations were correlated to macrohardness distribution analysis. The macro-Vickers was preferred over micro-Vickers due to existence of large (up to 80 μm) copper-based particles in the stir-zone. Under these conditions, the stir zone was not homogeneous and the large indent size of the macro-Vickers averaged out the inhomogeneities to obtain a bulk hardness value. The calculation of macrohardness distribution profile was carried out by an Instron Wolpert 4021 V-Testor (Norwood, MA, USA) (3000 gf for 15 s). Furthermore, the macrohardness values were correlated with a FSPed specimen without copper addition (AA5083 plate with three FSP passes without any Al–Cu intermetallic compound reinforcements).

## 3. Results and Discussion

### 3.1. Microstructural Characterization of the FSPed Specimens

#### 3.1.1. Optical Microscopy Results

**Figure 2:**
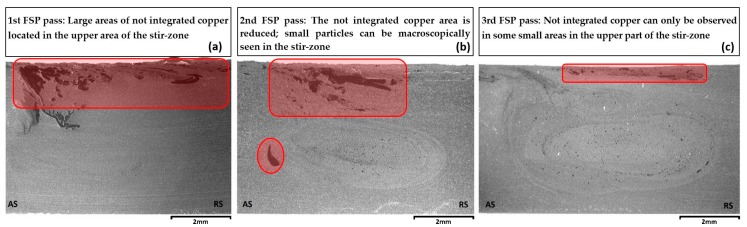
Macrographs of the cross-sections of Cu-reinforced FSPed 5083-H111 specimens with: (**a**) one, (**b**) two, (**c**) three FSP passes.

Figure 2 shows macrographs of cross-sections of the FSPed Cu-reinforced aluminum alloy with one, two and three FSP passes respectively. The dark areas are the copper that has not been integrated in the AA5083 stir zone during the FSP. It can be easily observed that the not-integrated copper areas tend to disappear during the subsequent FSP passes. More specifically, in the specimen with only one FSP pass, large copper areas are introduced in the upper area of the stir zone (Figure 2a). In the specimen with the two FSP passes, this area tends to get reduced, and small copper-based particles (copper particles that through diffusion processes have created different intermetallic layers) tend to appear in the stir zone (Figure 2b). In the specimen with the three FSP passes (Figure 2c), the dark not-integrated copper can only be observed in some small areas in the upper part of the stir zone (inside the Shoulder Affected Zone). Furthermore, many fine copper-based particles tend to appear well distributed inside the stir zone. Thus, the specimen with the three FSP passes was chosen for further microstructural investigation.

**Figure 3:**
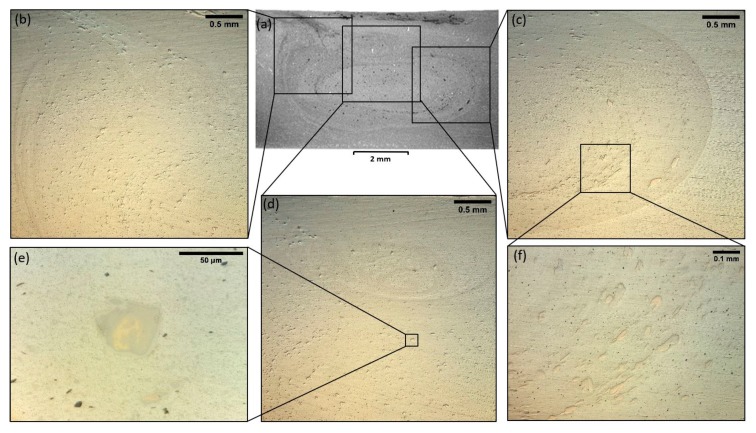
(**a**) OM macrograph of the Cu-reinforced FSPed 5083-H111 specimen with three FSP passes; (**b**) OM micrograph near the advancing side; (**c**) OM micrograph near the retreating side; (**d**) OM micrograph of the center of the stir zone; (**e**,**f**) OM micrographs of typical copper-based embedded particles at 1000× and 200× magnifications respectively.

Figure 3 illustrates optical microscopy images of the stir zone from the specimen with the three FSP passes. Overall, the stir zone is characterized by the absence of typical FSW/FSP defects (such us tunnel defects, void/wormhole defects, groove like defects and cracks). The applied parameters (tool rotational speed of 1000 rpm, transverse speed of 13 mm/min and three FSP passes) yield the required results, as they optimize material mixing and particle scattering. A small flow arm was formatted in the upper part of the stir zone (Figure 3a, red indication). The flow arm zone is found on the upper area of the weld and consists of material that is dragged by the shoulder from the retreating side of the weld, around the rear of the tool, and deposited on the advancing side [6]. 

Copper-based particles are found well distributed at the concentric circles of the weld nugget (Figure 3b–d). The particles have diameters ranging from 5 μm to 80 μm. Figure 3e,f depicts typical copper-based embedded particles at a higher magnification. Those particles appear to have layers of different colors that can be attributed to different intermetallic phases. Furthermore, most of the particles appear to be dimensionally oriented, following the material flow of the FSP process (Figure 3f).

#### 3.1.2. Scanning Electron Microscopy Results

**Figure 4:**
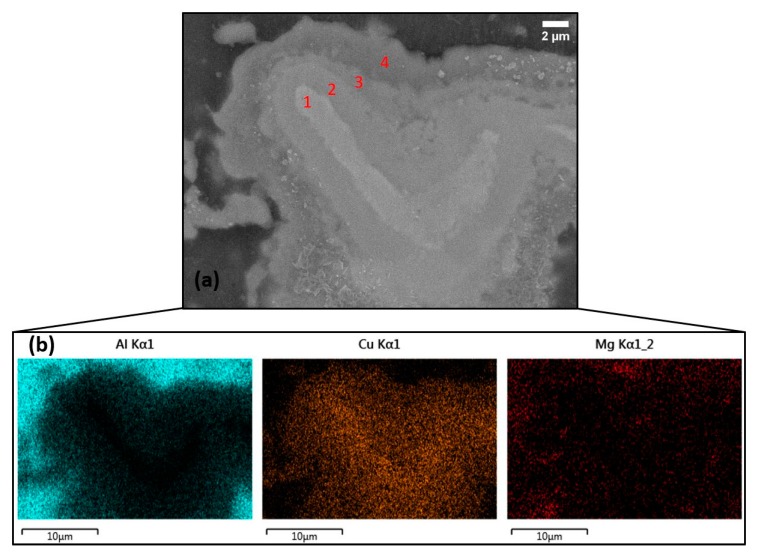
(**a**) Scanning electron image of typical copper-based embedded particles (20.0 kV SEI), (**b**) EDS mapping analysis.

A microstructural investigation using scanning electron microscopy (SEM with EDS) was also performed. Thus, Figure 4a shows a SEM image of a typical copper embedded particle located at the center part of the stir zone. As also observed from the optical microscopy images, the particle consisted of different layers. At least four discrete layers can be easily observed (Figure 4a, red numbers). In order to investigate the compositions of those layers, EDS mapping analysis was performed. Results show that each layer consisted of a different stoichiometric composition (Figure 4b). The darker the layer of the SEM image, the more aluminum concentrated within. The color differences between the layers is attributed to the differences in atomic weights caused by the different stoichiometric compositions. The closer the layer to the center of the particle, the more copper the intermetallic phase layer consisted of. The whiter layer/core at the center of the particle is mainly pure copper (the copper layer/core was mainly observed in large particles with diameters ranging from 30 to 80 μm). The copper particles, during the FSP process, due to the intense heat input and plastic deformation, tend to get enriched with aluminum atoms through interplanar diffusion–interfacial migration effects. Thus, different layers with different stoichiometric compositions are created. Furthermore, the dark area outside the particle mainly consisted of aluminum and magnesium, but small traces of copper can also be identified. This means that a small amount of copper has been diffused into the AA5083 FSPed matrix. Finally, magnesium, despite the low content, was also found diffused in all the layers of the particle. Small traces can be also found in the inner pure copper layer of the particle (Figure 4a, layer 1). This can be attributed to different elemental diffusion coefficients [21]. 

#### 3.1.3. Atomic Force Microscopy Results

**Figure 5:**
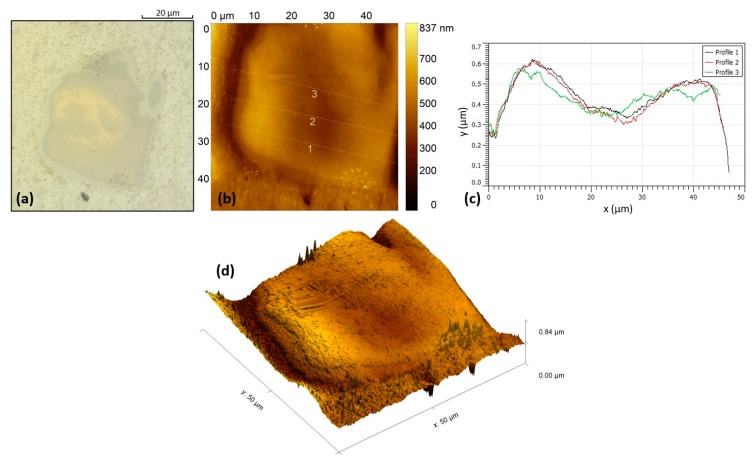
Copper-based embedded particle: (**a**) OM image; (**b**) AFM topographic image; (**c**) AFM linear height profiles; (**d**) AFM 3D view.

Atomic force microscopy (AFM) was performed on a typical layered particle. Figure 5a shows the optical microscopy image of the investigated particle at 1000× magnification. The particle has a diameter of about 50 μm and four different layers can be observed (layers with different colors). The brighter yellow layer at the center is the pure copper. Figure 5b illustrates the AFM 2D topographic image of the same particle. During the specimen’s metallographic grinding and fine polishing process, areas with higher rigidity/hardness tend to expel material at lower rates. Thus, the height differences of the layers, as can be better observed in the line profile plot of Figure 5c, can be attributed to different values of rigidity/hardness of the layers. The center of the particle that consists mostly of pure, soft copper is lower than the other layers that are characterized by hard, brittle and complex intermetallic phases. The height difference of each layer can be visually seen in the 3D AFM topographic image of Figure 5d.

#### 3.1.4. X-Ray Diffraction Analysis Results

**Figure 6:**
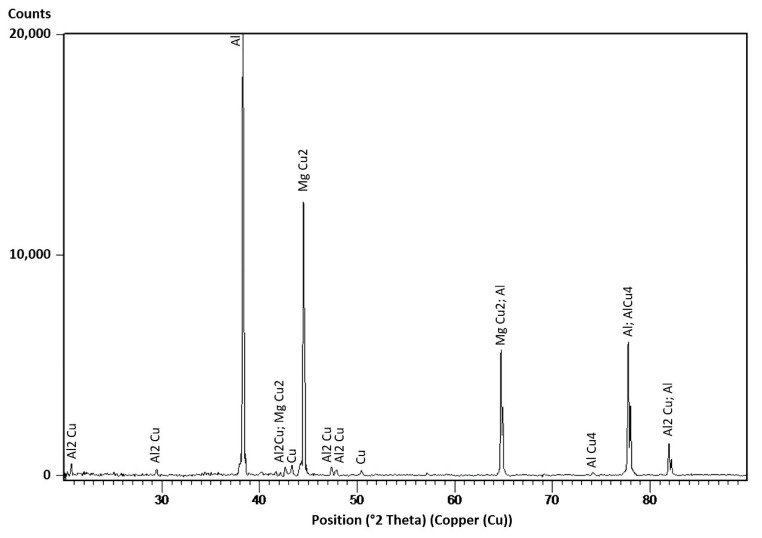
X-ray diffraction pattern of Cu-reinforced FSPed AA5083.

X-ray diffraction (XRD) analysis was utilized to identify the macrotextural evolution and the phases present in the FSPed Cu-reinforced three-pass specimen. As shown in Figure 6, after the three FSP passes, the small copper peaks still exist. This is attributed mainly to the pure copper core in the larger embedded particles (Figure 4). However, most of the copper particles had reacted with aluminum and magnesium and formed intermetallic phases during FSP. The main phases that were safely detected are: a face cubic centered (FCC) interstitial solid solution of aluminum matrix (α-Al), an interstitial solid solution of copper matrix (Cu) and an Al_2_Cu intermetallic phase (θ-Al_2_Cu). Furthermore, a AlCu_4_ intermetallic phase and a MgCu_2_ intermetallic phase were also detected. It should be mentioned that the X-ray diffraction analysis has a constraint in identifying phases below a bare minimum threshold volume fraction of 2%. Additionally, phases with particularly refined structures and heterogeneous distributions are difficult to safely distinguish and recognize. Thus, other phases could be present but not detected in the XRD results.

### 3.2. Macrohardness Distribution Analysis

**Figure 7:**
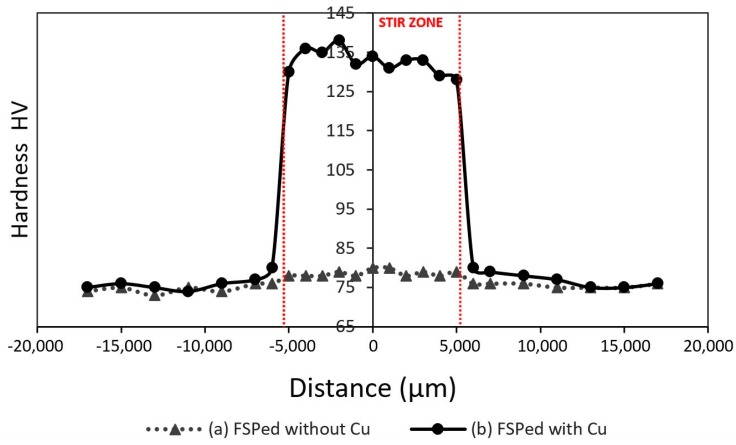
Macrohardness distribution profile of (**a**) FSPed specimen without Cu; (**b**) FSPed specimen with Cu.

Regarding the macrohardness distribution of the Cu-reinforced three-pass FSPed sample, the average hardness inside the stir zone reached up to 138 HV (Figure 7). The high increase in macrohardness values, compared to the base material (75 HV) and the FSPed sample without the copper addition (78 HV), is attributed mainly to the high presence of the copper-based embedded particles. Those particles consisted mainly of different complex and hard intermetallic layers. Thus, the stir zone with the particles embedded to the base metal matrix acts similarly to a metal matric composite (MMC) reinforced with ceramic particles. Another possible reason for the hardness increase is the diffusion of the copper in the AA5083 matrix. The diffusion of the reinforcing metal in the matrix could lead to a solid solution matrix that is mechanically tougher than the pure base metal matrix. Furthermore, the embedded particles and the diffused copper in the AA5083 matrix could act as barriers to the movement of dislocations, thereby increasing the hardness of the material. Finally, a very small increase of the hardness was observed in the thermo-mechanical-affected zone (TMAZ). This small increase can be attributed to copper diffusion from the stir-zone to the TMAZ, but safe conclusions cannot be obtained and further research is needed.

## 4. Discussion

Based on the present work, a very promising method for surface modification and surface hardening is presented. The main novelty of the suggested method lies in the fact that a copper strip can be easily integrated in the aluminum stir-zone, with the use of FSP, in te form of micron-sized particles, and thus modify the mechanical and physical properties of the weld. The copper particles, during the FSP process, due to the intense heat input and plastic deformation, tend to get enriched with aluminum atoms through interplanar diffusion–interfacial migration effects, and layers of different hard and complex phases are created in-situ. The implementation of a metal strip instead of powder minimizes the volume of reinforcing material needed, reduces the air inserted into the stir zone and introduces a more economical process of surface composite fabrication. The proposed process could have potential applications for manufacturing hybrid alloys. For example, materials that on one side are pure AA5083, and have very good corrosion resistance and moderate mechanical properties, on the other side (the cu reinforced side) have better mechanical properties with increased hardness and wear resistance. The possible applications of this method are of high interest in modern industries, and research is needed for the application of the method for different combinations of reinforcing and base metals. 

## 5. Conclusions

From the above-presented results, the following summarizing remarks can be drawn:

- The scope of this study was successful in producing a Cu-reinforced AA5083 composite stir-zone.
- The proposed process with the utilization of 1000 rpm rotational speed combined with 13 mm/min transverse speed and three sequential FSP passes resulted in almost complete integration of the copper strip in the stir zone.
- The integration was done mainly in the form of coper-based intermetallic particles, and secondly, by copper diffusion in the AA5083 matrix.
- Microstructural analysis showed that the copper-based particles were composed by layers of different stoichiometric compositions. At the center of the particles, a pure copper layer/core was observed (mainly in large particles with diameters ranging from 30 to 80 μm).
- The main aluminum-copper intermetallic phases that were probed from the XRD analysis were the Al_2_C and AlCu_4_.
- The macrohardness distribution inside the Cu-reinforced stir-zone was highly increased from 77 HV to 138 HV.
- The high increment in macrohardness values in the stir-zone, compared to the FSPed sample without the copper addition, is attributed mainly to the high presence of the Al–Cu intermetallic compounds, and secondly to the diffused copper in the AA5083 matrix.

## Figures and Tables

**Table 1:** Chemical composition of base material.

Element	Al	Si	Fe	Cu	Mn	Cr	Mg	Ti	Others Total
Weight %	94.42	0.11	0.26	0.01	0.62	0.09	4.45	0.01	0.03
